# Editorial: Outbreak oracles: how AI's journey through COVID-19 shapes future epidemic strategy

**DOI:** 10.3389/frai.2025.1636444

**Published:** 2025-06-13

**Authors:** Dmytro Chumachenko, Jasleen Kaur, Jake Y. Chen

**Affiliations:** ^1^Mathematical Modelling and Artificial Intelligence Department, National Aerospace University “Kharkiv Aviation Institute, ” Kharkiv, Ukraine; ^2^Faculty of Health, University of Waterloo, Waterloo, ON, Canada; ^3^Department of Biomedical Informatics and Data Science, The Informatics Institute, University of Alabama at Birmingham, Birmingham, AL, United States

**Keywords:** artificial intelligence, COVID-19, epidemic modeling, digital surveillance, pandemic preparedness, explainable AI, public health decision-making, data ethics

In the wake of the coronavirus disease 2019 (COVID-19) pandemic, the convergence of artificial intelligence (AI), Big Data, and *in silico* simulation has emerged as a cornerstone in pandemic surveillance and public health informatics. As early as January 2020, AI-driven tools, such as BlueDot, flagged unusual pneumonia clusters in Wuhan days before the World Health Organization (WHO) public alert (Brownstein et al., 2009). Meanwhile, platforms like CORD-19 were developed to aggregate SARS-CoV-2 literature for rapid machine-driven synthesis (Wang et al., 2020). By mid-2021, AI-based forecasting models were routinely incorporated into national response dashboards, demonstrating that algorithmic surveillance could anticipate hospitalization peaks with a lead time of up to 2 weeks (Institute for Health Metrics and Evaluation, 2019). This transformative period witnessed AI's potential to reshape public health strategies, emphasizing its significance in future epidemic preparedness. However, challenges such as data privacy, algorithmic bias, and unequal access to technological infrastructure persist as obstacles to global adoption. Addressing these limitations is essential to ensure the inclusive and ethical deployment of AI in public health.

The primary objective of this Research Topic is to collate groundbreaking research and critical reviews that highlight AI's contributions during the COVID-19 era and its implications for future epidemic strategies. We aim to foster a comprehensive understanding of the pivotal AI-driven methodologies in the pandemic response and how these innovations can be harnessed for future health crises.

The Research Topic includes original research, technology, code, as well as perspective papers. We received 16 submissions, 9 of which, after a careful review process, were accepted for publication in this Research Topic.

Drawing on Johns Hopkins surveillance data from 34 countries, a study by Nesteruk(a) evaluates how demographic structure and surveillance intensity shaped the late-phase contours. Regression analyses of 2022–2023 COVID-19 incidence, mortality, and case–fatality rates reveal median age as the primary determinant: older populations recorded higher numbers of detected cases and deaths per million. Testing density displayed a strong association with incidence, yet when insufficient, it exaggerated fatality ratios.

Song et al. introduce K-Track-COVID, an R Shiny dashboard that unifies government APIs, WorldPop demographics, and KDCA line lists to render fine-grained maps, regional time series, and SEIRD/SVEIRD scenario forecasts for all 17 South Korean provinces. Interactive controls enable users to filter dates, strata, and health-system indicators, while an embedded stochastic simulator generates animations and tabular projections 100 days ahead. Compared with the WHO, Johns Hopkins University (JHU), and the Centers for Disease Control and Prevention (CDC) dashboards, K-Track-COVID uniquely couples hotspot analytics with modifiable epidemiological models, offering a transferable template for data-driven preparedness beyond COVID-19.

Barmak et al. advance explainable AI by introducing a transition-matrix framework that maps high-dimensional embeddings onto clinician-defined features, thereby rendering model outputs transparent without retraining. The authors formalize the mapping as a pseudo-inverse solution and implement a visual analytics pipeline that scales across modalities. Validation on the Massachusetts Institute of Technology-Beth Israel Hospital Electrocardiogram (MIT-BIH ECG) arrhythmia and ACDC cardiac-MRI datasets yields Cohen's κ of 0.89 and 0.80, respectively, demonstrating strong alignment with expert annotations while preserving diagnostic accuracy.

A study by Oliveira et al. presents a cohort study spanning eight Brazilian hospitals (*n* = 421) to develop low-cost triage models for COVID-19. Using 28 collected admission variables, the authors construct seven data subsets to explore missing data strategies and train 27 classical algorithms, as well as dense neural networks, under Monte Carlo cross-validation. A random forest achieved 80% accuracy (AUC: 0.91), while an SVC yielded 87% PPV with minimal false positives. Dyspnea, general condition, SpO_2_, age, and urea emerged as key predictors, underscoring the potential of pragmatic AI deployment in resource-constrained settings.

Nesteruk's(b) study generalizes the classical SIR framework by partitioning infectious and removed compartments into registered and unregistered subgroups and introducing a visibility coefficient β, which yields a five-equation system whose analytical solutions facilitate accelerated parameter estimation. Applying the model to two pertussis waves in England (2023–2024) under full case ascertainment, he reproduces the cumulative and daily incidence curves with a 17% 4-month predictive error.

Melnykova et al. developed an AI pipeline for analyzing and correcting post-traumatic dysarthric speech in military patients. Using the TORGO corpus, they benchmarked a mel-spectrogram CNN against an MFCC-based LSTM, achieving accuracies of 94% and 91%, respectively. The CNN achieves higher precision and recall for healthy and low-severity classes, whereas the LSTM performs better in detecting severe cases. Feature-saliency analysis highlights spectral cues guiding rehabilitation. Ensemble modeling and data augmentation are proposed to generalize across accents and noise, underscoring AI's promise for scalable speech therapy.

A study by Chaikovsky et al. investigates whether subtle quantitative alterations in serial 12-lead electrocardiograms (ECGs) predict outcomes in severe COVID-19. Among 26 intensive care unit (ICU) patients (six of whom died), the authors computed 240 waveforms and HRV metrics, summarized by a composite U-score. They paired them with NEWS and SpO_2_ for cluster and CART modeling. T-wave singular value decomposition (SVD), R-wave amplitude (lead II), and Q-wave amplitude (lead I) proved to be the most discriminative, yielding a three-split decision tree that classified survival with 96% cross-validated accuracy, highlighting ECG micrometrics as a practical bedside prognostic tool.

Lyimo et al. surveyed 76 Environmental Health Officers across Morogoro, Ilala, and Dodoma to assess their readiness for ML-based forecasting of waterborne diseases. With a 66% response rate, respondents displayed moderate ICT competence yet limited AI literacy: only 54% had previously encountered AI/ML and 64% rated their familiarity as low. While the majority recognized ML's potential to improve outbreak prediction, they flagged infrastructure gaps, poor data quality, and skill shortages as barriers.

The perspective article by Huaiyan et al. offers a comprehensive view on how digital technologies will redefine infectious disease practice in the aftermath of COVID-19. Surveying bioinformatics, AI, big data analytics, nanotechnology vaccines, blockchain, and telemedicine, the authors map converging trends in prevention, early diagnosis, therapy, and supply–chain governance, while highlighting the ethical imperatives of data sovereignty and algorithmic bias.

In the future, we urge the community to align AI outbreak tools with the Findability, Accessibility, Interoperability, and Reusability (FAIR) Data Principles and the WHO's Preparedness Strategy (2023), ensuring that models are not only performant but interoperable and equitable across low-resource settings (Wilkinson et al., 2016). By building on the lessons of COVID-19, where transparency, open data, and cross-disciplinary collaboration proved critical, the field should coalesce around a shared framework that accelerates innovation and trust in future AI-powered epidemic responses.
